# Enhanced Development of Skeletal Myotubes from Porcine Induced Pluripotent Stem Cells

**DOI:** 10.1038/srep41833

**Published:** 2017-02-06

**Authors:** Nicholas J. Genovese, Timothy L. Domeier, Bhanu Prakash V. L. Telugu, R. Michael Roberts

**Affiliations:** 1 C.S. Bond Life Sciences Center, University of Missouri, Columbia, MO 6521, USA; 2 Department of Medical Pharmacology and Physiology, University of Missouri, Columbia, MO 65211, USA; 3 Department of Animal and Avian Sciences, University of Maryland, College Park, MD 20742, USA; 4 Animal Bioscience and Biotechnology Laboratory, USDA ARS, Beltsville, MD 20705, USA

## Abstract

The pig is recognized as a valuable model in biomedical research in addition to its agricultural importance. Here we describe a means for generating skeletal muscle efficiently from porcine induced pluripotent stem cells (piPSC) *in vitro* thereby providing a versatile platform for applications ranging from regenerative biology to the *ex vivo* cultivation of meat. The GSK3B inhibitor, CHIR99021 was employed to suppress apoptosis, elicit WNT signaling events and drive naïve-type piPSC along the mesoderm lineage, and, in combination with the DNA methylation inhibitor 5-aza-cytidine, to activate an early skeletal muscle transcription program. Terminal differentiation was then induced by activation of an ectopically expressed *MYOD1*. Myotubes, characterized by myofibril development and both spontaneous and stimuli-elicited excitation-contraction coupling cycles appeared within 11 days. Efficient lineage-specific differentiation was confirmed by uniform NCAM1 and myosin heavy chain expression. These results provide an approach for generating skeletal muscle that is potentially applicable to other pluripotent cell lines and to generating other forms of muscle.

One of the main driving forces for pursuing studies on porcine pluripotent stem cells has been to create tissue precursors *in vitro* that might be used to explore engraftment procedures in an animal model with close physiological and morphological similarity to the human^1^. Unfortunately, true embryonic stem cells from agriculturally important large animals, including the pig, have proven difficult to generate, and piPSC, which are readily created, have only rarely been used for directed differentiation purposes^2^. As far as we are aware, for example, no studies to date have described differentiation of piPSC along the mesoderm lineage and overcoming the barrier to myogenesis, despite the widespread interest in using the pig in modeling infarction and cardiovascular disease, muscle regeneration and muscle biology^3^,^4^,^5^. In addition, skeletal muscle tissue is the major constituent of pork, and piPSC are a potential source of self-renewing, skeletal muscle progenitors for creating cultured meat and other livestock products^6^. Thus, the molecular control mechanisms directing porcine skeletal muscle development have considerable implications for medicine, agriculture and food technology.

From the earliest stages of embryonic development through terminal differentiation, skeletal myogenesis is directed by CTNNB1 (β-catenin) and GSK3B (glycogen synthase kinase-3β). Phosphorylation at the targeted motif of CTNNB1 by GSK3B marks the protein for proteasomal degradation, while hypophosphorylated CTNNB1 can either stably associate with the cell membrane or form complexes with lymphoid enhancer binding factor (LEF1, sometimes known as TCF) to activate an expression suite of target genes within the nucleus^7^. Multiple signaling pathways converge at GSK3B to modulate CTNNB1 activity during embryonic development and myogenesis. WNT signaling, for example, can activate CTNNB1 by GSK3B inactivation^8^. During epiblast gastrulation, early mesodermal patterning is mediated by the transcription factor T (BRACHYURY)^9^. Like T, expression of a premyogenic transcription factor, PAX3, is directed by WNT signaling^10^,^11^,^12^,^13^ and is CTNNB1-activated^14^,^15^,^16^. PAX3 is expressed initially in the pre-segmented paraxial mesoderm, and later increases in the somite^17^ where, in combination with other factors, a NOTCH signaling checkpoint prevents precocious myotome maturation^18^. Subsequent WNT signaling^19^,^20^,^21^ directs sequential expression of the functionally overlapping^22^, but distinct myogenic regulatory factors (MRFs), MYF5 and MYOD1, signifying commitment to the skeletal muscle lineage. WNT and IGF signaling^23^,^24^,^25^,^26^,^27^, coupled with repression of activin receptor-like kinase (ALK) activity^28^,^29^, support terminal differentiation, marked by cell-cycle exit and cell fusion into multinucleated skeletal myofibers capable of generating mechanical force by contraction. To evaluate the potential of piPSC to give rise to mesodermal lineages and skeletal muscle, in particular, we activated developmental pathways characterized in these human and mouse studies to support lineage specification of piPSC to skeletal muscle *in vitro,* and assessed the capacity of the lineage-specified precursor cells to differentiate terminally into contractile myotubes.

## Results

### CHIR99021 attenuates apoptosis following ground-state withdrawal

**Figure 1:**
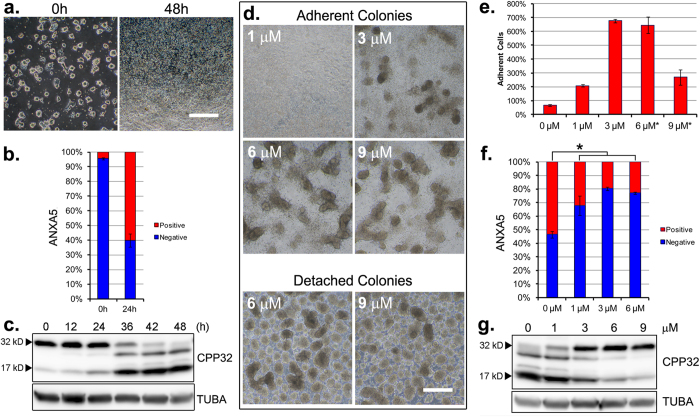
An axis of apoptosis and differentiation is modulated by CHIR99021. **(a)** Morphology of ground-state piPSC colonies cultured for three days from single cells in renewal conditions (RM-1, *left panel*) and following 48 hour (h) differentiation (RM-2, *right panel*). **(b)** ANXA5 (annexin V) labeling of apoptotic cells prior to, and following, 24 h differentiation. Error bars represent *n* = 3 experimental replicates ± SD. **(c)** Western blot detection of full-length CPP32 (~32 kD procaspase 3a) and the large cleaved fragment (~17 kD cleaved-caspase 3a) prior to (0 h) and following (12–48 h) differentiation. **(d)** Phase contrast images of adherent (*upper panels*) and detached colonies (lower panels), following 48 h differentiation + 1–9 μM CHIR99021, as indicated. **(a**,**d)** Scale bars, 500 μM. **(e)** Percent cell adhesion following 48 h differentiation without, as shown in **(a)**, *right panel*, or with 1–9 μM CHIR99021, as shown in **(d).** *viable cells within detached colonies unaccounted. Error bars represent *n* = 3 experimental replicates ± SEM. **(f)** ANXA5 labeling of apoptotic cells following 24 h differentiation ± CHIR99021, as indicated. Error bars represent *n* = 3 experimental replicates ± SEM. *Significance (*P* < 0.05) determined by *t*-test. **(g)** Western blot detection of full-length and cleaved CPP32 following 42 h differentiation ± CHIR99021, as indicated. **(c,g)** TUBA detected as an internal protein loading control. Western blots were cropped for clarity. Examples of uncropped blots are found in Supplementary Figure 9.

A naïve piPSC line derived from the inner cell mass of a porcine blastocyst that expressed *POU5F1* and *KLF4* reprogramming genes from a doxycycline (DOX)-inducible promoter (Supplementary Fig. 1) was employed throughout^30^. As with naive mouse embryonic stem cells (ESC)^31^, naïve piPSC culture was supported by a medium (Renewal Medium, RM-1; Supplementary Table 1) containing CHIR99021/PD032591/PD173074 (inhibitors of GSK3B/MEK/FGF signaling) and LIF under a 5% O_2_, 5% CO_2_, 90% N_2_ atmosphere. To identify conditions that might permit differentiation from the ground state, piPSC colonies were dissociated to single cells and then sub-cultured on a poly-D-lysine, laminin and gelatin (PLG) substratum in RM-1 containing DOX in order to maintain expression of the two reprogramming genes, which are essential for self-renewal in this cell line. By three days, the cells had formed colonies (Fig. 1a, *left panel*). To support differentiation, the RM-1 renewal medium was replaced with a differentiation medium (DM-1; Table S1) formulated without DOX, LIF and CHIR99021/PD032591/PD173074. The cultures were transferred to a 20% O_2_, 5% CO_2_, 75% N_2_ atmosphere. Extensive cell death was observed over the two days following the transition (Fig. 1a, *right panel*). Increased cell-surface labeling by ANXA5 (Fig. 1b, Supplementary Fig. 2) and onset of CPP32 cleavage (Fig. 1c) indicated that cell death was linked to an intrinsic apoptosis pathway.

Next, retention of CHIR99021 was tested as an approach to prevent the massive amount of cell death encountered in its absence. In these experiments, colonies were cultured in differentiation medium (DM-1) supplemented with CHIR99021. Improved cell survival and cell adhesion was observed over the full range (1–9 μM) of CHIR99021 concentrations tested. All concentrations between 3–9 μM supported formation of attached, rounded colonies, but, at the higher (6–9 μM) concentration cell aggregates break free from the colonies to form floating structures resembling embryoid bodies (Fig. 1d). The improved adhesion to the matrix observed between 3 to 6 μM CHIR99021 (Fig. 1e), plus the reduced presence of cell-surface ANXA5 (Fig. 1f, Supplementary Fig. 3) and intracellular, cleaved CPP32 (Fig. 1g), indicated that CHIR99021 had attenuated apoptosis.

### CHIR99021 activates WNT signaling events during piPSC differentiation

**Figure 2:**
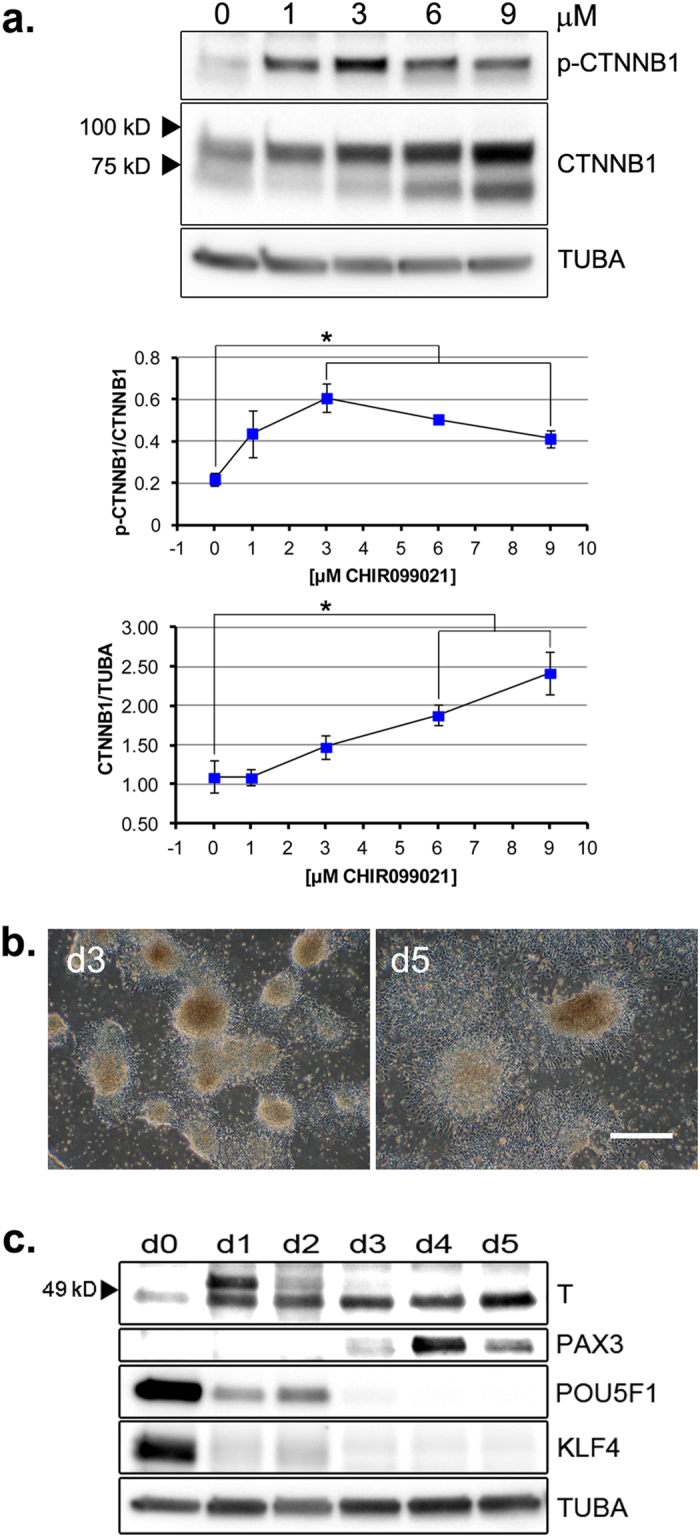
CHIR99021 stabilization of CTNNB1 and primary differentiation of piPSC colonies. **(a)** *Upper Panel:* Western blot detection of CTNNB1 and p-CTNNB1 (total and phospho-S33,37,T41 β-catenin, respectively) following one day differentiation ± CHIR99021, as indicated. *Middle Panel:* ratios of p-CTNNB1/CTNNB1 bands. *Lower Panel:* ratios of CTNNB1/TUBA bands. *Middle and Lower Panels*: Error bars represent *n* = 3 experimental replicates ± SEM. *Significance (*P* < 0.05) determined by *t*-test. **(b)** Adhesion and outgrowth morphology following transfer of day (d) 2 (Fig. 1d) detached colonies differentiating in 6 μM CHIR99021/DM-1 to PLM-coated culture dishes, for one (d3, *left panel*) or three (d5, *right panel*) additional days. Scale bar, 500 μM. **(c)** Western blot analysis of T, PAX3, POU5F1 and KLF4 expression in ground-state (d0) and differentiating cultures (d1–d5) during the regimen described in (**b**). (**a,c**) TUBA detected as an internal protein loading control. Western blots were cropped for clarity. Examples of uncropped blots are found in Supplementary Figure 9.

To determine whether CHIR99021/DM-1 activated canonical WNT signaling events, relative phospho- and total CTNNB1 was monitored after transfer of piPSC to DM-1 culture medium with and without CHIR99021. As anticipated, CHIR99021 exposure (3–9 μM) resulted in a dose-dependent increase of CTNNB1. CHIR99021 exposure also increased CTNNB1 phosphorylation at the GSK3B-targeted motif, but there appeared to be less accumulation of phospho-CTNNB1 at concentrations above 3 μM (Fig. 2a).

CTNNB1 stabilization and morphological changes observed during CHIR99021/DM-1 culture suggested that mesodermal determination might have been initiated. To investigate this possibility, embryoid body-like structures from day 2 of differentiation (Fig. 1d) were transferred to poly-D-lysine, laminin and Matrigel (PLM) substratum in CHIR99021/DM-1, where they adhered and formed outgrowths (Fig. 2b). Expression of ground-state pluripotency markers POU5F1 and KLF4, and mesodermal markers T (BRACHYURIA) and PAX3, were monitored over six days, from ground-state colony formation (day 0), to detachment of differentiating colonies (days 1–2), to adherence and outgrowth of detached colonies (days 3–5). Expression of POU5F1 and KLF4 were down-regulated to basal levels by day 3. An antibody against human T detected two ~49 kD bands within a migration range expected for the protein. The faster migrating isoform was detected at day 0, increased by day 2, and was maintained through day 5. Expression of a higher M_r_ isoform was observed on days 1 and 2, and then declined, the significance of which is unclear. Transient expression of PAX3 was observed on days 3–5 (Fig. 2c). Together, these features suggested that the cultures had attained a mesodermal state primed for myogenic induction.

### Efficient myogenic induction of piPSC

**Figure 3:**
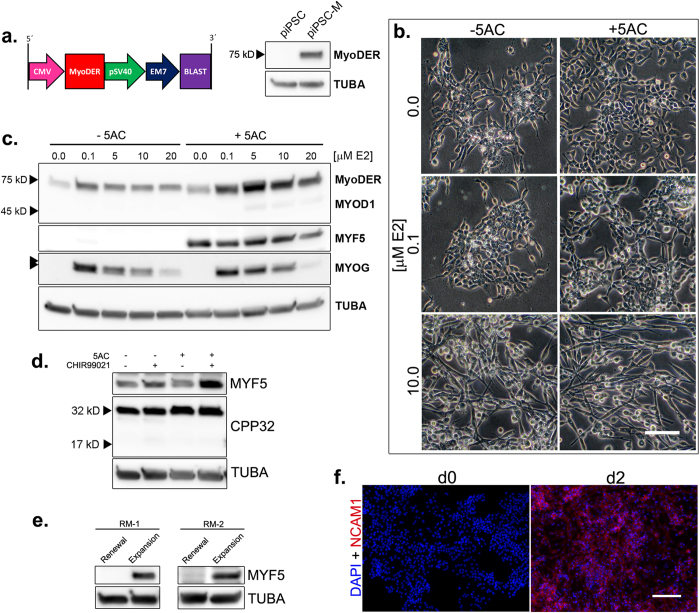
Activation of endogenous MRFs by 5AC and ectopic MYOD1. **(a)** piPSC modification by an integrated MyoDER expression cassette. *Left panel*: blasticidin (BLAST)-selectable MyoDER expression cassette. Arrows and boxes indicate promoter and gene sequences, respectively. *Right panel*: Western blot detection of MyoDER in the unmodified piPSC and the expression cassette-modified piPSC-M. MyoDER was detected with an antibody raised against a mouse MYOD1 peptide. **(b)** Morphology of differentiating piPSC-M cultures following expansion and differentiation regimens shown in Supplementary Figure 5a. Scale bar, 100 μM. **(c)** Western blot detection of MYOD1 antigens, MYF5 and MYOG following piPSC-M expansion and differentiation regimens shown in Supplementary Figure 5a. *Double arrowheads*: partially resolved MYOG isoforms. **(d**,**e)** Determinants of MYF5 activation: Western blot analyses. **(d)** Detection of MYF5 and CPP32 following piPSC differentiation regimens, as shown in Supplementary Figure 5b. **(e)** Detection of MYF5 following three days of piPSC-M culture in alternate (RM-1, RM-2) renewal or expansion conditions. **(a,c,d,e)** TUBA detected as an internal protein loading control. Western blots were cropped for clarity. Examples of uncropped blots are found in Supplementary Figure 9**. (f)** Immunofluorescent detection of nuclei (DAPI) and NCAM1 (AlexaFluor 568) in differentiating cultures prior to (d0, *left panel*) and following (d2, *right panel*) myogenic induction (RM-1→IM-1). Scale bar, 150 μM.

Commitment to the skeletal muscle lineage is directed through transcription program activation by MYF5, MYOD1 or both. Ectopic MYOD1 expression or exposure to 5-aza-cytidine (5AC) has been reported to activate the endogenous myogenic program in certain cell lines^32^,^33^. To impart ectopic MYOD1 activity, the parental piPSC line was modified by integration of a selectable cassette expressing an estrogen receptor (*ESR1*) ligand-binding domain insertion within a *MYOD1* open reading frame (*MyoDER*) (Fig. 3a). The expressed MyoDER protein can be activated conditionally by 17β-estradiol (E2) (Supplementary Fig. 1)^34^.The modified piPSC (piPSC-M) expressed a ~75 kD MyoDER fusion protein (Fig. 3a), and, in the absence of the E2 agonist, continued to form compact, refractive colonies characteristic of the parental colonies (Supplementary Fig. 4).

Dosages of 5AC and E2, alone and in combination, were then screened for toxicity and effects on cell morphology. Although 5AC exposure reduced colony growth across the dosage range tested (Supplementary Fig. 4), a 250 nM concentration was tolerated sufficiently well to provide viable cells for subsequent differentiation steps, and was selected for use in future procedures. Since a PLM substratum supported cell adhesion by differentiating piPSC and expression of PAX3 (Fig. 2b,c), we conjectured that it might also support skeletal muscle differentiation in CHIR99021/DM-1. Therefore, 3 μM CHIR99021, a concentration that supported cell adhesion, survival (Fig. 1d–g), and CTNNB1 phosphorylation (Fig. 2a) was added to the DM-1 to facilitate differentiation in the presence of 5AC and/or E2. As shown in Supplementary Figure 5a, piPSC-M were first expanded in RM-1 ± 5AC on a PLM substratum for three days, followed by culture in CHIR99021/DM-1 supplemented with combinations of 5AC and E2 for two additional days.

E2 (5–20 μM) induced a bipolar morphology characteristic of skeletal myocytes. 5AC enhanced the E2 effects, but, on its own, failed to induce this phenotype (Fig. 3b). Both 5AC and E2 increased MyoDER protein abundance in the cells (Fig. 3c). Expression of the endogenous MYOD1 protein (~45 kD) was not observed. MYF5 expression was activated by 5AC, independently of E2. In contrast, MYOG expression was activated by E2, independently of 5AC. However, relative MYOG abundance in the E2-induced cultures was reduced by increasing the E2 concentration. MYOG migrated as two molecular weight forms, with the faster migrating band favored by the combination of 5AC and E2. Together, these data suggested that, in combination with 5AC, induction of MYOD1 by10 μM E2 might be optimal for skeletal muscle lineage induction. Therefore, a medium containing 10 μM E2 and 3 μM CHIR99021 in DM (induction medium; IM Supplementary Table 1) was subsequently used for myogenic induction.

Our observation that CHIR99021/DM-1 failed to promote MYF5 expression in the absence of 5AC (Fig. 3c) suggested synergistic roles of 5AC and CHIR99021. To examine these roles, unmodified piPSC were expanded for three days on PLM substratum in renewal medium (RM-2, Supplementary Table 1) ±5AC and differentiated for two days in differentiation medium (DM-2, Supplementary Table 1) with combinations of CHIR99021 and 5AC, as shown in Supplementary Figure 5b. A sequential RM-2 expansion and DM-2 differentiation regimen supported detectable, but low MYF5 abundance. In the absence of CHIR99021, 5AC failed to increase MYF5. Rather, the highest MYF5 abundance occurred in medium supplemented with both 5AC and CHIR99021 (Fig. 3d). CPP32 cleavage associated with apoptotic cell death was not elicited under these conditions (compare to Fig. 1a,c).

We next examined whether MYF5 was expressed during the initial three-day expansion regimen. The piPSC were cultured for three days in renewal medium on PLG substratum (renewal conditions) or in renewal medium on PLM substratum plus 5AC (expansion conditions). For comparison, the procedure was conducted in two different renewal media, RM-1 and RM-2 (Supplementary Table 1). As expected, MYF5 was not detected in either renewal condition. However, it was detected in both expansion conditions (Fig. 3e), indicating early myogenic determination in the presence of DOX, LIF and CHIR99021/PD032591/PD173074, prior MyoDER induction by E2.

After a sequential three-day expansion and two-day induction course with 5AC exposure on PLM substratum, piPSC-M progeny exhibited myocyte-like morphology, enhanced ectopic MYOD1 expression, and elevated expression of endogenous MYF5 and MYOG. This regimen led to uniform onset of expression of a skeletal muscle cell-surface glycoprotein, NCAM1, during, but not before, the two-day induction course (Fig. 3f). Collectively, these results demonstrate efficient piPSC specification to the skeletal muscle lineage.

### Terminal differentiation of lineage-specified piPSC-derived progeny

**Figure 4:**
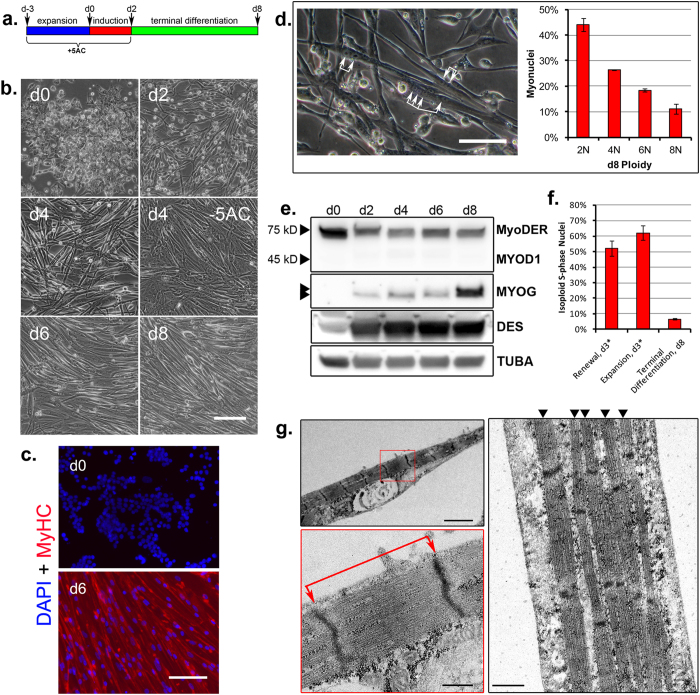
Terminal myogenesis by piPSC progeny. **(a**) 11-day culture regimen, with terminal differentiation. **(b)** Myotube morphology and conformation during a RM-2→IM-2→TDM regimen. Scale bar, 125 μM. **(c)** Day 0 (d0) and 6 (d6) cultures were stained for nuclei (DAPI) and MyHC class proteins (AlexaFluor 568). **(d)** Myotube multinucleation. *Left panel*: terminal differentiation morphology, day 4 (RM-1→IM-1→TDM). Bracketed arrows mark multiple nuclei within single myotubes. *Right panel*: myonuclei distribution within day 8 (d8) di- and polyploid myotubes. Error bars represent *n* = 3 experimental replicates ± SD. **(c,d)** Scale bars, 100 μM. **(e)** Western blot analyses. MYOD1 antigen, MYOG and DES detection, days 0–8. *Double arrowhead*: partially resolved MYOG isoforms. TUBA detected as an internal protein loading control. **(f)** Cell-cycle exit and terminal differentiation. Western blots were cropped for clarity. Examples of uncropped blots are found in Supplementary Figure 9. **(d,f)** Populations labeled and enumerated as shown in Supplementary Figure 7. Error bars represent *n* = 3 experimental replicates ± SD. *d3 expansion is equivalent to day 0 (d0) induction, as shown in **(a)**. **(g)** Transmission Electron Microscopy, day 6 myotubes. Myofibrils and sarcomeric subunits within the myotube sarcolemma. *Left panels*: Single myofibril within the sarcolemma (*upper left panel*) and an enlarged inset (red box, *lower left panel*) to visualize the sarcomere structure. Red bracketed arrows mark the sarcomere length, from Z-line to Z-line. *Right panel*: multiple, parallel myofibrils within the sarcolemma, marked by arrowheads. Scale bars: *upper left panel*, 2.0 μM; *lower left panel*, 0.4 μM; *right panel*, 0.5 μM.

To assess the terminal differentiation potential of lineage-specified cells, cultures were maintained for up to six additional days in terminal differentiation medium (TDM) (Fig. 4a). The TDM was formulated without E2, which acted as a pleiotropic antagonist of MYOG expression (Fig. 3c), and with CHIR99021, IGF-1 and an ALK inhibitor, A83–01, to support terminal differentiation (Supplementary Table 1). Expression of DES (desmin), a muscle intermediate filament structural protein, was detected at low levels by day 0, and was up-regulated progressively throughout the induction and terminal differentiation steps (Fig. 4e). By day 4, multinucleated cells with elongated, refractive morphologies characteristic of myotubes were evident (Fig. 4b,d). Otherwise equivalent differentiation regimens excluding 5AC treatment during the expansion and induction steps failed to yield such characteristic morphologies (Fig. 4b, Supplementary Fig. 6). By day 6, myotubes exhibited regional anisotropic patterning (Fig. 4b), uniform expression of myosin heavy chain (MyHC) class proteins (Fig. 4c), and myofibrils with well-defined sarcomeric subunits (Fig. 4g). The average sarcomere length observed was 1.76 ± 0.23 μM SD, *n* = 21, which is within the resting range documented for metazoan skeletal muscle^35^. Following induction, MYOG expression was maintained through day 6 and was strongly up-regulated by day 8. Endogenous MYOD1 was not detected over the differentiation course (Fig. 4e). Spontaneous contractions by random myotube populations were observed beginning on day 4 during the terminal differentiation period, a process that led to partial myotube detachment from the substratum (Supplementary Video 1). By day 8, most cells had fused into polyploid myotubes (Fig. 4d, Supplementary Fig. 7), and most nuclei had stopped replicating DNA, indicating that cells had exited from the cell cycle and had committed to terminal differentiation (Fig. 4f, Supplementary Fig. 7).

### Spontaneous and stimuli-responsive excitation-contraction coupling of piPSC-derived myotubes

**Figure 5:**
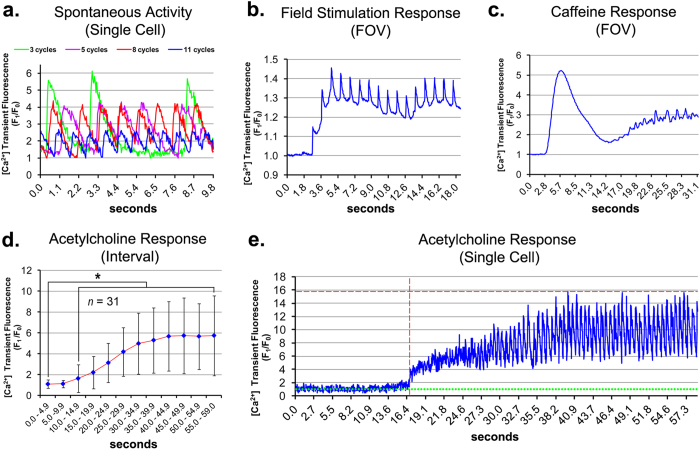
Spontaneous and stimuli-induced Ca^2+^ transient activity in piPSC-derived myotubes. Myotubes were loaded with the Ca*2* + indicator dye Fluo-4 AM at T_0_ and imaged by high-speed confocal fluorescence microscopy. Dynamic Ca^2+^ signaling events within single cells or an entire field-of-view (FOV) were recorded and plotted. Ca^2+^ signal is shown as F_1_/F_0_; F_1_ represents the variable raw signal during the image sequence; F_0_ represents the constant baseline signal. For **(a)**, F_0_ corresponds to the lowest signal during the respective trace. For **(b–e)**, F_0_ corresponds to the respective signal recorded at 0.0 s (seconds). **(a)** Spontaneous Ca^2+^ transient activity in regionally active day 6 myotubes. Representative single-cell traces show simultaneous activities in four myotubes coinciding in the same FOV. **(b)** Ca^2+^ transient response by mechanically quiescent day 6 myotubes to 1.0 Hz field stimulation. **(c)** Ca^2+^ transient response by mechanically quiescent day 6 myotubes to 10 mM caffeine perfusion. **(b,c)** The response trace shows total signal from the respective FOVs over the duration indicated. **(d)** Mean 5 s Ca^2+^ transient interval response following perfusion of mechanically quiescent day 7 myotubes with 100 nM acetylcholine; *n* = 31 ± SD. *Significance (*P* < 0.05) determined by *t*-test. **(e)** Ca^2+^ transient response to acetylcholine perfusion by a single myotube represented in **(d)**. The vertical dashed red line marks the threshold activation lag at F_1_/F_0_ = 3.0. The horizontal dashed red line marks the maximum signal amplitude (Amp_*MAX*_). The horizontal dotted green line marks the baseline signal (F_1_/F_0_ = 1).

In skeletal muscle, excitation-contraction coupling (ECC) occurs via voltage-dependent Ca^2+^ release into the cytosol, where dihydropyridine receptors sense depolarization at the plasma membrane and open adjacent ryanodine receptor Ca^2+^ release channels of the sarcoplasmic reticulum by allosteric interaction^36^. To characterize the contractile physiology of the differentiating myotubes, intracellular Ca^2+^ signaling was monitored during terminal differentiation. Asynchronous oscillations of intracellular Ca^2+^ transient cycles of varied frequency and amplitude were observed in spontaneously contracting populations (Fig. 5a, Supplementary Video 2). Aside from these populations, myotubes remained mechanically quiescent in the absence of external stimuli. Electrical field stimulation (1.0 Hz) elicited both a synchronized Ca^2+^ transient response corresponding to the stimulation frequency, and elevated cytosolic Ca^2+^ during field stimulation intervals. During stimulation, the average cyclic period (peak-to-peak) recorded was 1.0 ± 0.02 seconds SD, *n* = 15 (Fig. 5b, Supplementary Video 3). Electrically-induced Ca^2+^ transients were observed in the absence of extracellular Ca^2+^, indicating a voltage-dependent Ca^2+^ release as the underlying mechanism for the phenomenon (*n* = 2, data not shown), characteristic of skeletal muscle^37^. Caffeine, an agonist that triggers cytosolic Ca^2+^ release through ryanodine receptors, caused an initial high-amplitude, synchronous Ca^2+^ response followed by asynchronous Ca^2+^ oscillations (Fig. 5c, Supplementary Video 4). Application of acetylcholine, a native agonist of skeletal muscle contraction, also induced significant Ca^2+^ signaling within myotube cultures (Fig. 5d, Supplementary Video 5). Single-cell analysis revealed that acetylcholine exposure initiated both a gradual rise in cytosolic Ca^2+^ and cyclical Ca^2+^ oscillations (Fig. 5e). Individual acetylcholine responses varied in stimulation lag, amplitude, activity and magnitude (Supplementary Fig. 8), accounting for the high variance in the aggregate data (Fig. 5d).

## Discussion

Our hybrid approach to induce skeletal muscle lineage specification by piPSC employs a coordinated application of CHIR99021, 5AC and an ectopically expressed MYOD1. Although each of these factors has been reported to enhance skeletal muscle derivation from pluripotent progenitors *in vitro*, a combinatorial application provides multiple advantages. A previous study that targeted GSK3B inhibition during mouse ESC differentiation by LiCl exposure included an intermediate cell sorting step to enrich myogenic progenitors prior to terminal differentiation^38^. More recent studies that used CHIR99021 or the GSK3B inhibitor (2′*Z*,3′*E*)-6-bromoindirubin-3′-oxime (BIO) to direct human iPSC differentiation reported sarcomeric development following 21 to 40-day differentiation regimens at varied efficiencies^39^,^40^,^41^,^42^. In our study, both enhanced MYOG expression and sarcomeres were observed within eleven days (Fig. 4e,g), demonstrating an accelerated, enriched development of NCAM^+^/MyHC^+^ cells without cell sorting.

One study has described an enrichment of skeletal muscle markers in differentiating human ESC populations by 5AC treatment^43^. However, this enrichment was incomplete. Unlike the results of our study (Figs 3c–f, and 4c,e), differentiating populations failed to express MYF5, DES, MyHC or NCAM1, *in vitro*. Results from multiple studies that have used an ectopic MYOD1 to derive skeletal muscle from pluripotent progenitors have varied. An early study demonstrated that, although ectopic MYOD1 was sufficient to activate MYOG expression in differentiating mouse ESC, an intermediate embryoid body step was required to support expression of MYF5 and MyHC. Moreover, the efficiency of skeletal muscle differentiation was low^44^. A later study reported myogenic differentiation directed by a Tet-Off *MYOD1* cassette-modified mouse ESC line, but neither expression of endogenous MYOD1, nor endogenous MYF5, were confirmed^45^. Studies that have reported the conversion of human ESC and iPSC to skeletal muscle by ectopic MYOD1 expression in pluripotent cells or their derivatives differentiated *in vitro*^46^,^47^,^48^,^49^ have failed to demonstrate the activation of endogenous MYF5 expression.

In this study, we applied the self-renewal capacity of a piPSC line to allow indefinite expansion of myogenic progenitors^30^. This cell line exhibits certain characteristics of a recently described F-class mouse iPSC^50^, including a renewal dependence upon DOX-enforced reprogramming gene expression (not shown), and a low-threshold 5AC tolerance (Supplementary Fig. 4). In the absence of DOX, LIF, PD032591 and PD173074, cell survival and differentiation were maintained conditionally by CHIR99021 (Figs 1 and 2), thereby elucidating a fundamental role for GSK3B in cell death during differentiation by naïve piPSC. This study further revealed an unexpected role for 5AC in promoting expression of MYF5 rather than endogenous MYOD1, as previously reported during myogenic conversion of other cell types^33^. Expression of the *MYF5* gene is regulated directly by CTNNB1/LEF1 binding and transcriptional co-activation of the *MYF5* epaxial enhancer^51^. In our study, phosphorylation and stabilization of CTNNB1 during piPSC differentiation was directed by CHIR99021 (Fig. 2a). Moreover, expression of the MYF5 protein was either reduced or not detected (Fig. 3c–e) in the absence of 5AC or CHIR99021 exposure. We speculate that in combination, 5AC and CHIR99021 may activate *MYF5* gene expression by stabilizing CTNNB1 interaction with a hypomethylated *MYF5* epaxial enhancer. During the ontological progression from embryonic progenitors to skeletal muscle, MYF5 expression precedes MYOD1 activity^52^. In chronological agreement, MYF5 and its transcription target, *DES*^53^, were expressed during our differentiation course (Fig. 4a) by the third day of expansion culture (Figs 3e and 4e), and prior to the activation of ectopic *MYOD1* by E2.

A faster-migrating MYOG isoform (Fig. 3c), possibly corresponding to the active, hypophosphorylated protein^54^, was enriched by 5AC exposure. Moreover, without 5AC exposure, myotube development was impaired (Fig. 4b, Supplementary Fig. 6), implicating a role for 5AC in directing a myogenic identity prior to terminal differentiation. Like myotubes from somatic myogenic progenitors^55^, piPSC-derived myotubes exhibited spontaneous contractile activities (Supplementary Videos 1, 2). Additionally, these myotubes responded to electrical and chemical stimuli in a manner consistent with the physiological signaling pathways actuating skeletal muscle excitation-contraction coupling, *in vivo* (Fig. 5b–e, Supplementary Videos 3,4,5). Together, these data provide molecular, cytological and physiological lines of evidence supporting the generation of skeletal muscle during our differentiation regimen.

The methods describe combinatorial approaches to optimize lineage specification of piPSC to skeletal muscle. They demonstrate production of contractile porcine myotubes over an expedited course with efficiency and fidelity in serum-free culture media. These results signify piPSC as a promising alternative to somatic myogenic progenitors, such as satellite cells, which have limited self-renewal properties for various applications that use porcine cells^1^, and furthermore, validate the use of piPSC for production of porcine skeletal muscle at a potentially unlimited scale. Further studies will be required to customize and extend this approach for additional species and cell lines.

## Methods

### Cell culture

Derivation of the DOX-inducible piPSC line (O2K) used in this study has been described previously^28^. For naïve piPSC renewal, cultures were maintained on poly-D-lysine^a^, mouse laminin^b^, and porcine gelatin^a^ (PLG)-coated culture surfaces under 5% atmospheric O_2_ in renewal medium (RM) consisting of serum replacement, neurobasal medium^c^ and DMEM-F12^d^ combined at a 1:1 ratio, 1X non-essential amino acids^a^, 0.5X GlutaMAX^c^, 0.1 mM β-mercaptoethanol^a^, 0.1 mg/mL protease-free bovine serum albumin^e^, 2 μg/mL doxacycline hyclate (DOX)^f^, 10 ng/mL human leukemia inhibitory factor (LIF)^g^, 3 μM CHIR99021^b^, 0.8 μM PD032591^h^ and 0.1 μM PD173074^a^. Ground-state piPSC colonies were enzymatically dissociated and passaged every three days. For general differentiation, day 3 piPSC colonies were cultured under 20% atmospheric O_2_ in a basal differentiation medium (DM) formulated as RM, but with β-mercaptoethanol, DOX, LIF, CHIR99021, PD032591 and PD173074 omitted. For myogenic expansion, cultures were maintained as renewal cultures, except that piPSC were cultured on poly-D-lysine, laminin and Matrigel^i^ (PLM)-coated culture surfaces, and RM was supplemented with 250 nM 5-aza-cytidine (5AC)^a^ each day for three days prior to myogenic induction. For myogenic induction, the RM of day 3 myogenic expansion cultures was replaced with induction medium (IM), and cultures were differentiated for two additional days with 5AC supplementation. IM was prepared as DM, but additionally supplemented with 3 μM CHIR99021 and 10 μM 17β-estradiol (E2)^a^. For terminal differentiation of skeletal muscle lineage-specified cultures following induction, IM was replaced with terminal differentiation medium (TDM) and differentiating cells were cultured for up to six additional days. TDM was prepared as IM, except that E2 was excluded, and the medium was formulated with 100 ng/mL human IGF-1^j^ and 4 μM A83–01^k^. 15% KnockOut Serum Replacement (KSR)^c^ or a combination of 0.5X N-2 Supplement^c^ + 0.5X B-27 Supplement without Vitamin A^c^ were alternately substituted as serum replacements. Culture media formulations are summarized in Supplementary Table 1.

### piPSC modification

The MyoDER DNA sequence was PCR amplified from the pBabeMDER plasmid^l^ and recombined into the pLentiCMVBlast lentiviral destination vector^l^ by using a pENTR/D-TOPO entry vector^c^. Pseudovirus prepared from supernatants of 293FT cells co-transfected with the modified lentiviral vector, pMD2.G envelope plasmid^l^ and the psPAX2 packaging plasmid^l^ was used for transduction of the piPSC. Stably-transduced piPSC were selected with 10 μg/mL blasticidin^m^ for four days and 15 μg/mL for two additional days. The modified piPSC were designated ‘piPSC-M’ and maintained as the unmodified piPSC, except to avoid unscheduled induction of the MyoDER protein, RM used to culture the piPSC-M was formulated with phenol red-free neurobasal^c^ and DMEM-F12^c^ basal media.

### Immunodetection and flow cytometry

For Western blotting, cell lysates were normalized to 50 μg total protein/lane, resolved by 5–15% gradient SDS-PAGE and transferred to a PVDF membrane. Membranes were probed with antibodies against CPP32 (#9662)^n^, PAX3 (Pax3)°, POU5F1 (#sc-5279)^p^, KLF4 (#sc-20691)^p^, CTNNB1 (#8480)^n^, phospho-CTNNB1 (#9561)^n^, MYOD1 (#sc-304)^p^, MYF5 (#sc-302)^p^, MYOG (F5D)°, DES (#sc-14026)^p^ and TUBA (#A01490)^q^. PVDF membranes were developed with SuperSignal West Dura Extended Duration Substrate^r^ and chemiluminescent images were captured with a Fujifilm LAS-300 CCD camera. To stabilize relative phospho-CTNNB1 to levels sufficient for detection, phosphatase and proteasome activities were inhibited by exposing the cultures to 50 nM calyculin A^n^ and 30 μM MG132^n^ for three hours prior to cell lysate preparation. Densitometry data were obtained by using ImageJ image quantification software (version 1.40 g). The AlexaFluor 488 Annexin V/Dead Cell Apoptosis Kit^3^ was used for ANXA5 labeling of apoptotic cells. To label replicating DNA, live cells were exposed 10 mM 5-ethynyl-2′-deoxyuridine (EdU)^c^ for two hours and then suspended in cold ethanol. Integrated EdU was fluorophore-conjugated by using the Click-iT Plus AlexaFluor 647 Flow Cytometry Assay Kit^c^. Total DNA was labeled overnight on ice with 20 μg/mL propidium iodide^c^ solution. ANXA5, EdU and propidium iodide labeling data were collected by means of an Accuri C6 flow cytometer running CFlow software (version 1.0.202.1) and data were processed by FlowJo flow cytometry analysis software (version 10). For immunofluorescent imaging, cells were cultured on PLM-coated glass coverslips and bound primary antibodies were labeled with AlexaFluor 568-conjugated goat anti-mouse secondary antibody^c^. For NCAM1 detection, coverslips were fixed in 4% paraformaldehyde and labeled with primary antibody 5.1H11°. For detection of myosin heavy chain (MyHC) class proteins, coverslips were fixed in −20 °C methanol and labeled with a pan-MyHC reactive primary antibody (MF 20)°. Immunolabeled coverslips were mounted on microscope slides in Vectasheild mounting medium with DAPI^s^ and images were captured with an Olympus IX70 inverted microscope.

### Ca^2+^ transient analyses

For live-cell Ca^2+^ transient imaging, piPSC were differentiated into myotubes on PLM-coated glass coverslips. The following steps were conducted at room temperature. Coverslips were washed with physiological saline solution (PSS; 1 mM CaCl_2_, 135 mM NaCl, 5 mM KCl, 1 mM MgCl_2_, 10 mM glucose and 10 mM HEPES pH 7.4) and loaded with 10 μM Fluo-4 AM^c^ Ca^2+^ indicator for 10 min and washed for ~50 min with PSS. While submerged in PSS, coverslips were transferred to a custom stage and fluorescence images (XYt mode) were obtained by using the resonance scanhead of a Lecia SP5/MP confocal microscope at 27.56 frames/s. Field stimulation (1.0 Hz, 2 ms pulse duration, ~20 volts), and perfusion with caffeine^a^ or acetylcholine^a^ were introduced immediately following (~1 s) initiation of the image sequence capture course. Data were processed and quantified using ImageJ image quantification software.

^a^Sigma-Aldrich, St. Louis, MO; ^b^Stemgent, Cambridge, MA; ^c^Life Technologies, Grand Island, NY; ^d^HyClone Laboratories, Logan, UT; ^e^Jackson ImmunoResearch Laboratories, Inc., West Grove, PA; ^f^MP Biomedicals North America, Solon, OH; ^g^EMD Millipore, Billerica, MA; ^h^Cayman Chemical Company, Ann Arbor, MI; ^i^Corning Incorporated Life Sciences, Tewksbury, MA; ^j^Shenandoah Biotechnology, Inc., Warwick, PA; ^k^Tocris Bioscience, Bristol, UK; ^l^Addgene, Cambridge, MA; ^m^InvivoGen USA, San Diego, CA; ^n^Cell Signaling Technology, Inc., Danvers, MA; °Developmental Studies Hybridoma Bank, Iowa City, IA; ^p^Santa Cruz Biotechnology, Inc., Dallas, TX; ^q^GenScript USA Inc., Piscataway, NJ; ^r^Pierce Biotechnology, Inc., Rockford, IL; ^s^Vector Laboratories, Inc., Burlingame, CA. Unless otherwise noted, materials were used in accordance with the supplier’s provided specifications.

## Additional Information

**How to cite this article:** Genovese, N. J. *et al*. Enhanced Development of Skeletal Myotubes from Porcine Induced Pluripotent Stem Cells. *Sci. Rep.* **7**, 41833; doi: 10.1038/srep41833 (2017).

**Publisher's note:** Springer Nature remains neutral with regard to jurisdictional claims in published maps and institutional affiliations.

## Supplementary Material

Supplementary Figures

Supplementary Video S1

Supplementary Video S2

Supplementary Video S3

Supplementary Video S4

Supplementary Video S5
