# Supplementary material for: Enhanced Development of Skeletal Myotubes from Porcine Induced Pluripotent Stem Cells
Source: Sci Rep. 2017 Feb 6;7:41833. doi: 10.1038/srep41833 (PMC5292944; doi:10.1038/srep41833)
Supplement: Supplementary Figures [file srep41833-s1.pdf]

# Enhanced Development of Skeletal Myotubes from Porcine Induced Pluripotent Stem Cells

Nicholas J. Genovese[1], Timothy L. Domeier[2], Bhanu Prakash V. L. Telugu[3,4], R. Michael Roberts[1*]

[1]C.S. Bond Life Sciences Center, University of Missouri, Columbia, MO 65211

[2]Department of Medical Pharmacology and Physiology, University of Missouri, Columbia, MO 65211

[3]Department of Animal and Avian Sciences, University of Maryland, College Park, MD 20742

[4]Animal Bioscience and Biotechnology Laboratory, USDA ARS, Beltsville, MD 20705

*Correspondence and requests should be addressed to R. Michael Roberts, 240b Bond Life Sciences Center, University of Missouri, Columbia, MO 65211. Email: robertsrm@missouri.edu

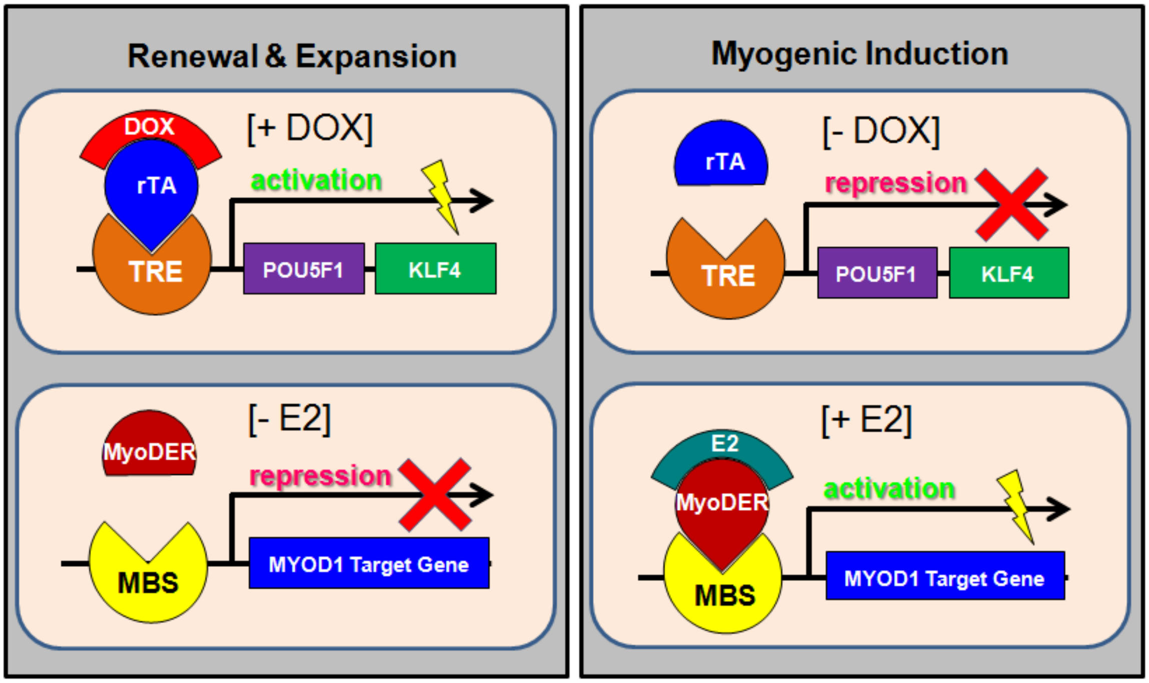


**Supplementary Figure 1.** *Double-switch* transcription network regulation in piPSC-M. Legend: DOX (doxycycline); E2 (17β-estradiol); TRE (tetracycline responsive element); rTA (tetracycline transactivator); MBS (MYOD1 binding site). *Left*: +DOX/-E2 conditions facilitate renewal and myogenic expansion. *Right*: -DOX/+E2 conditions support myogenic induction.

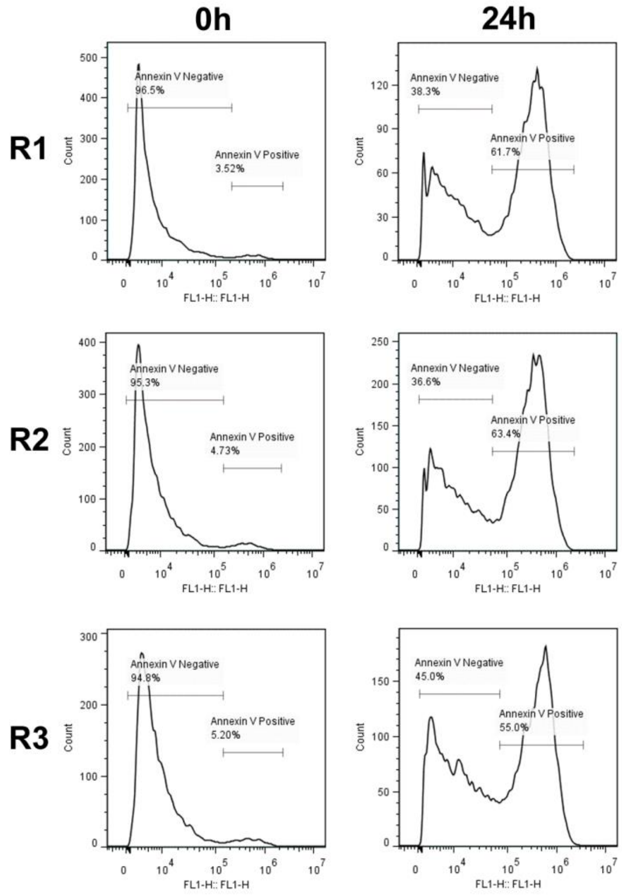


**Supplementary Figure 2.** Flow cytometry analyses of ANXA5 (annexin V) labeling prior to (0h) and following (24h) one-day piPSC colony transfer from RM-1 to DM-1, shown in **Figure 1b**. ANXA5 populations were partitioned at the minimum count interval between adjacent low and high ANXA5-labled population peaks (AlexaFluor 488, FL1-H), and designated negative or positive, respectively. Results from three replicate experiments (R1-3) shown.

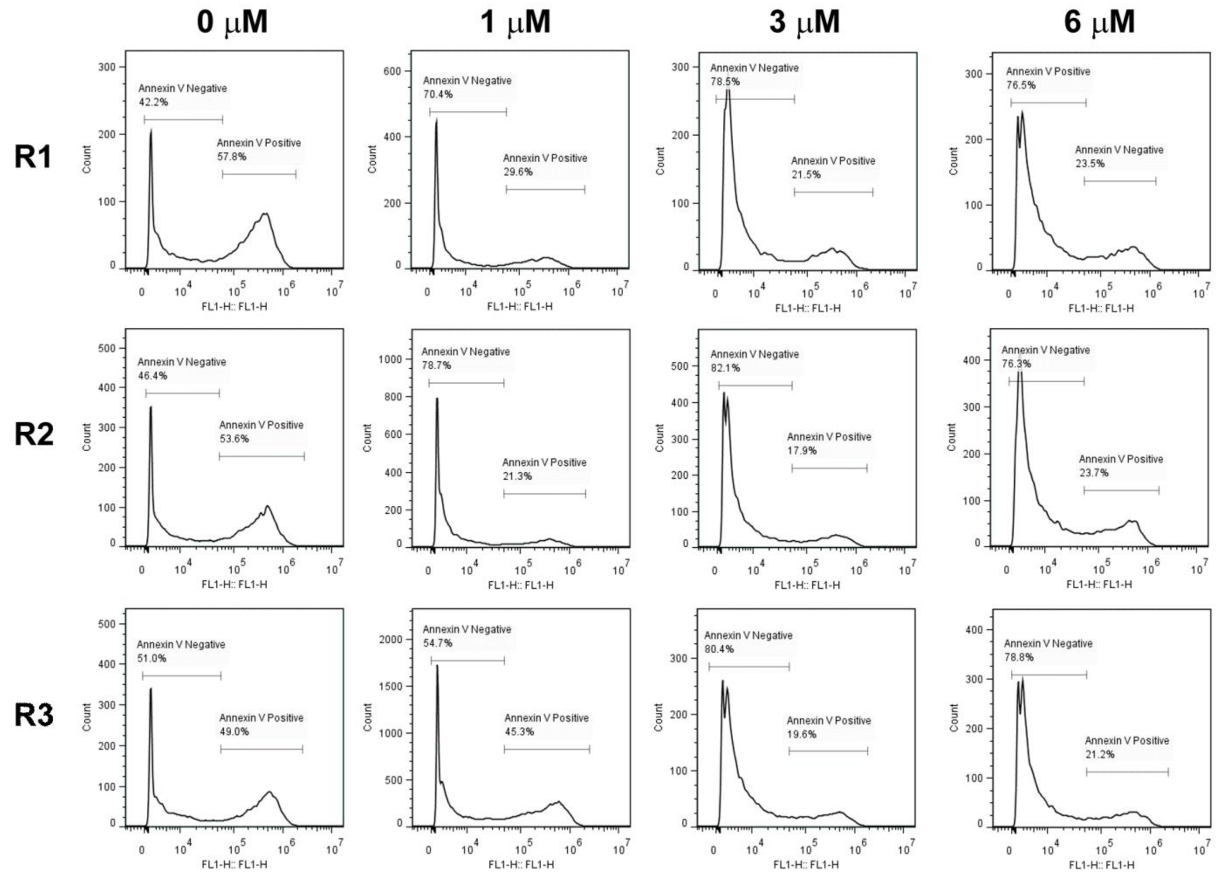


**Supplementary Figure 3**. Flow cytometry analyses of ANXA5 labeling following one-day differentiation culture of piPSC colonies ±CHIR99021**,** shown in **Figure 1f**. ANXA5 (annexin V) high and low-labeled populations were partitioned using identical gating (AlexaFluor 488, FL1-H) and designated positive or negative, respectively. Results from three replicate experiments (R1-3) shown.

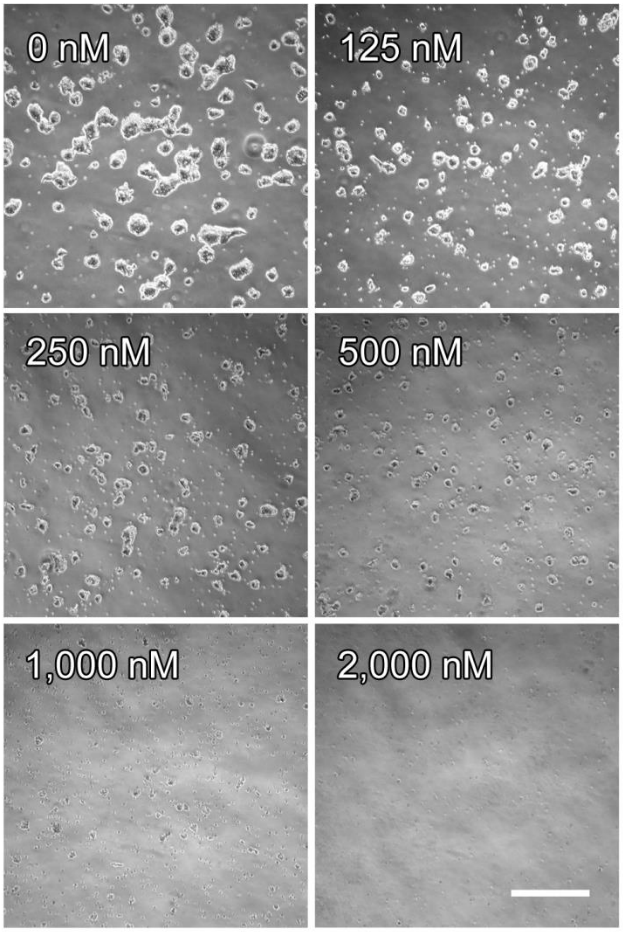


**Supplementary Figure 4.** piPSC-M colony morphology and dose-dependent response to 5AC exposure. piPSC-M were cultured in renewal conditions (RM-1) for three days from single cells in the absence (0 nM) or presence (125 – 2,000 nM) of 5AC. Scale bar, 500 μM.

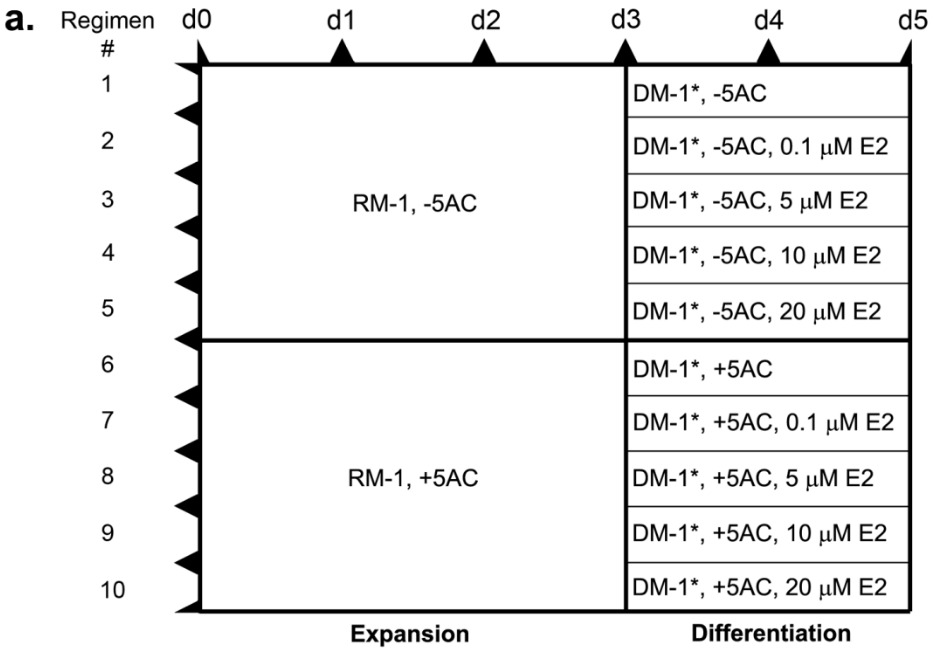


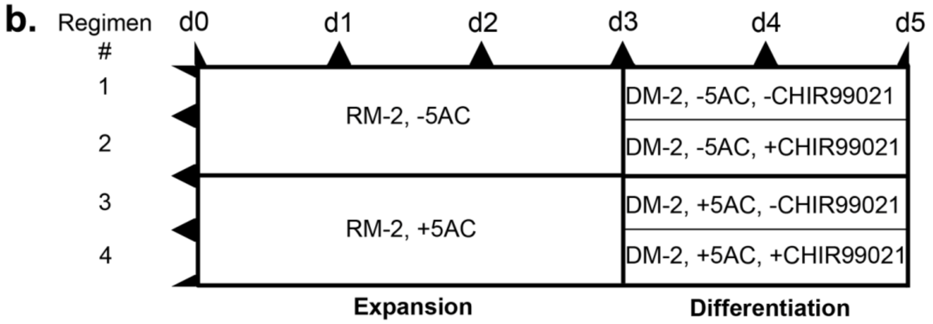


**Supplementary Figure 5.** Experimental expansion and differentiation regimens. **(a)** Culture regimens corresponding to the experiments shown in **Figure 3b**, **c**. *DM-1 formulations were prepared with 3 μM CHIR99021 and phenol red-free basal culture media. **(b)** Culture regimens corresponding to the experiments shown in **Figure 3d**.

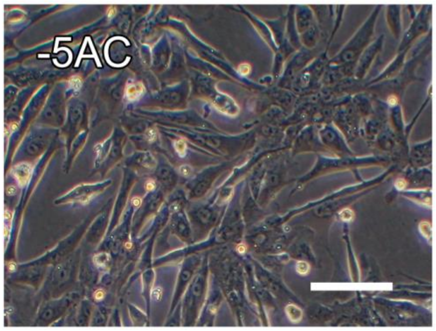


**Supplementary Figure 6.** Terminal differentiation culture morphology -5AC, day 4. Compare to **Figure 4b** (d4, *-5AC panel*) and **Figure 4d** (*left panel*). piPSC-M were differentiated according to the regimen shown in **Figure 4a**, except that 5AC exposure was omitted during the expansion and induction steps (as **Figure 4b**, d4 -5AC panel). As **Figure 4d** (*left panel*) N-2 & B-27 were substituted for KSR serum replacement during expansion and induction (RM-1→IM-1→TDM). Scale bar, 100 μM.

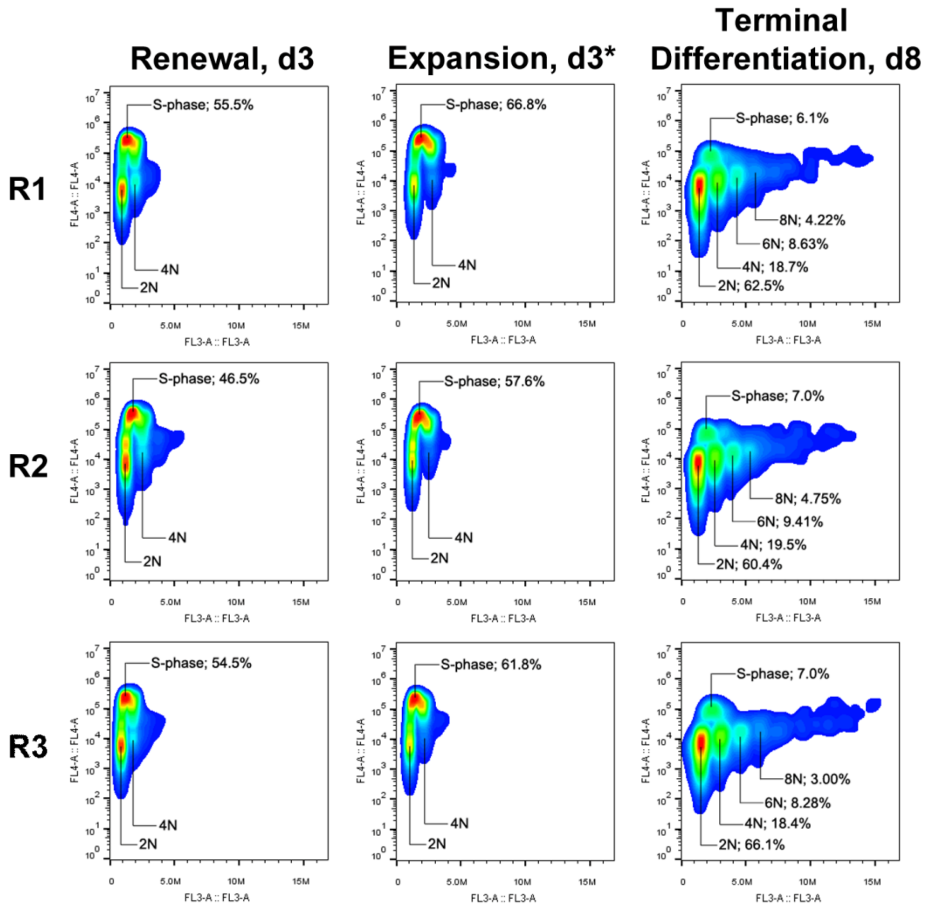


**Supplementary Figure 7.** Renewal, expansion and terminal differentiation culture DNA content analysis by flow cytometry. Heat map distribution plots of replicating (FL4-A) and total (FL3-A) DNA content labeled by EdU and propidium iodide, respectively, in piPSC-M and differentiating progeny. S-phase, 2N, 4N, 6N and 8N populations are annotated with their corresponding distribution frequencies at their population maxima. Populations are partitioned at the interval minima, on a density scale from blue (lowest) to red (highest). Results from three replicate experiments (R1-3) shown. Aggregate S-phase distributions are represented in **Figure 4f**. Percentage myonuclei distributions within cells are represented in **Figure 4d** (*right panel*), with S-phase populations excluded. Total myonuclei were determined by multiplying the ploidy myonuclei distribution percentage by the theoretical nuclei count and summing total nuclei. Myonuclei distributions were plotted as the percentage of nuclei associated with the respective ploidy. *Day 3 (d3) expansion is equivalent to day 0 (d0) induction, as shown in **Figure 4a**.

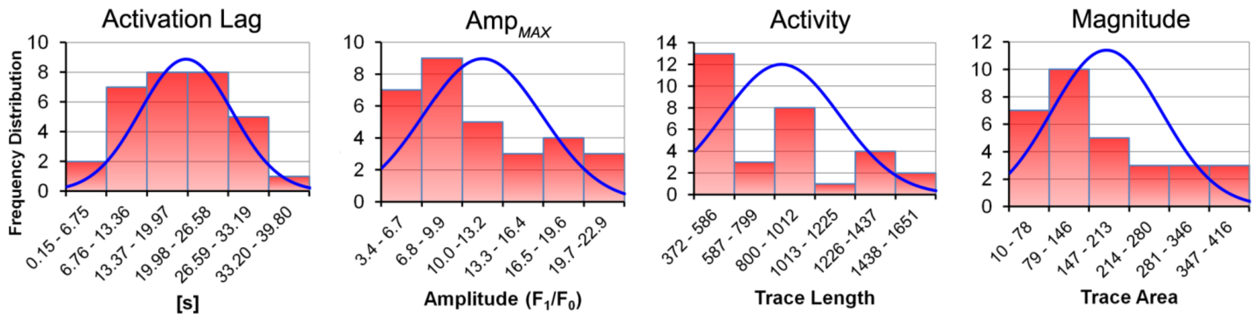


**Supplementary Figure 8.** Acetylcholine response variation. $Ca^{2+}$ transient response metrics, as shown in **Figure 5e**, were categorized (from *left* to *right*) by threshold activation lag at $F_1/F_0$ = 3.0, maximum signal amplitude ($Amp_{MAX}$), activity, and magnitude for $n$ = 31 individual myotube responses, collectively represented in **Figure 5d**. The response magnitude represents the summed areas bounded by the $Ca^{2+}$ response trace and the baseline signal. Activity is shown as the $F_1/F_0$ trace length. Activity and magnitude are represented as arbitrary units. Bars indicate the myotube distribution per interval. Curves depict the normal distribution for the categorical mean and SD.

**Supplementary Figure 9a to f:**
Examples of uncropped Western blots.

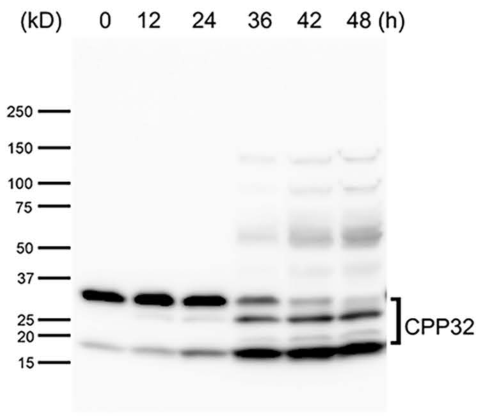


**9a.** Example of CPP32 uncropped Western blot, from Figure 1C.

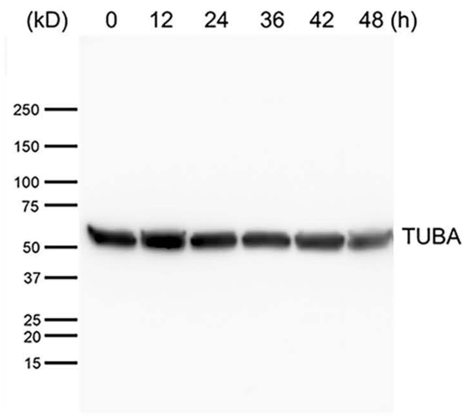


**9b.** Example of TUBA uncropped Western blot, from Figure 1C.

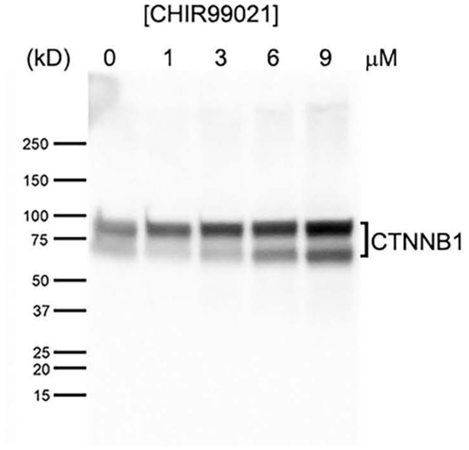


**9c.** Example of CTNNB1 Western blot, from Figure 2A.

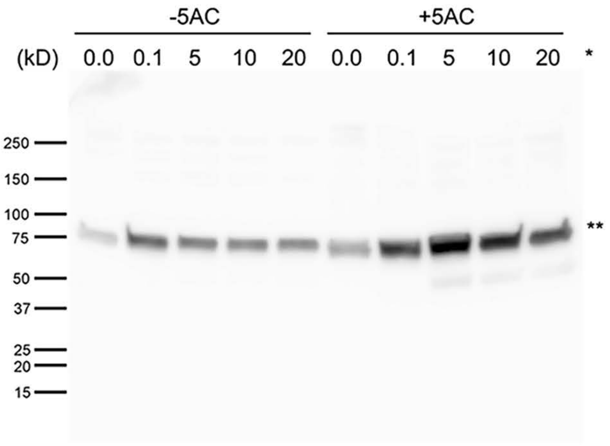


* [μM E2]
** MyoDER

**9d.** Example of MYOD1 Western blot, from Figure 3C.

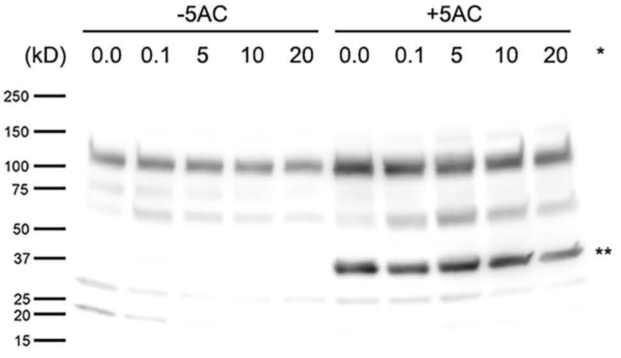


* [μM E2]
** MYF5

**9e.** Example of MYF5 Western blot, from Figure 3C.

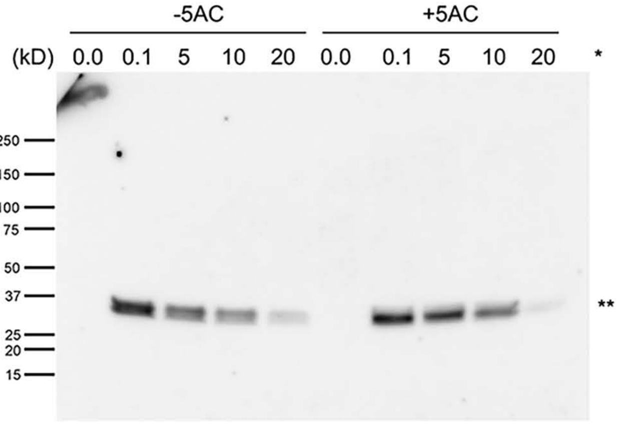


* [μM E2]
** MYOG

**9f.** Example of MYOG Western blot, from Figure 3C.

| Culture Medium | Serum Replacement | DOX, LIF, PD032591 & PD173074 | CHIR99021 | E2 | IGF-1, A83-01 | Atmospheric $O_2$ |
|---|---|---|---|---|---|---|
| **RM-1** | N-2 & B-27 | + | + | - | - | 5% |
| **RM-2** | KSR | + | + | - | - | 5% |
| **DM-1** | N-2 & B-27 | - | - | - | - | 20% |
| **DM-2** | KSR | - | - | - | - | 20% |
| **IM-1** | N-2 & B-27 | - | + | + | - | 20% |
| **IM-2** | KSR | - | + | + | - | 20% |
| **TDM** | N-2 & B-27 | - | + | - | + | 20% |

**Supplementary Table 1.** Formulations of culture media. Distinguishing components of renewal (RM), differentiation (DM), induction (IM) and terminal differentiation (TDM) media formulations summarized for comparison. Detailed formulations of respective media are described in *Results* and *Methods*. Briefly, media RM were used for renewal and expansion cultures; media DM were used for MyoDER-independent differentiation; media IM were used for MyoDER-directed differentiation following expansion; TDM was used for supporting myotube development from lineage-specified cells following induction. *Media were equilibrated at the designated oxygen tension for at least 1h prior to being used for culture under the respective $O_2$ condition. *Note:* 5AC was added directly to certain RM, DM and IM following culture medium replacement as described in *Results* and *Methods,* and is not included in the formulations listed above.

**Supplementary Video 1.** Spontaneous contraction. Phase-contrast video of regionally active, day 6 myotubes shown in real time, during a (RM-2→IM-2→TDM) regimen.

**Supplementary Video 2.** Spontaneous $Ca^{2+}$ transients. $Ca^{2+}$ transient image sequences of regionally active, day 6 myotubes were captured, pseudo-colored using a heat map depicting low (blue) to high (red→white) cytosolic $Ca^{2+}$ signal, and shown in real time.

**Supplementary Video 3.** $Ca^{2+}$ transient response to field stimulation. Following stimulation of regionally quiescent day 6 myotubes, $Ca^{2+}$ transient image sequences were captured, pseudo-colored using a heat map representing low (blue) to high (red→white) cytosolic $Ca^{2+}$ signal, and shown in real time.

**Supplementary Video 4.** $Ca^{2+}$ transient response to caffeine perfusion. Following perfusion of regionally quiescent day 6 myotubes, $Ca^{2+}$ transient image sequences were captured, pseudo-colored using a heat map representing low (blue) to high (red→white) cytosolic $Ca^{2+}$ signal, and shown in real time.

**Supplementary Video 5.** $Ca^{2+}$ transient response to acetylcholine perfusion. Following perfusion of regionally quiescent day 6 myotubes, $Ca^{2+}$ transient image sequences were captured, pseudo-colored using a heat map representing low (blue) to high (red→white) cytosolic $Ca^{2+}$ signal, and shown in real time.
